# Agreement between physicians and parents in rating functional ability of children with juvenile idiopathic arthritis

**DOI:** 10.1186/1546-0096-5-23

**Published:** 2007-12-11

**Authors:** Elena Palmisani, Nicoletta Solari, Angela Pistorio, Nicolino Ruperto, Clara Malattia, Stefania Viola, Antonella Buoncompagni, Anna Loy, Alberto Martini, Angelo Ravelli

**Affiliations:** 1 Pediatria II, Istituto di Ricovero e Cura a Carattere Scientifico G. Gaslini, Genova, Italy; 2 Servizio di Epidemiologia e Biostatistica, Direzione Scientifica, Istituto di Ricovero e Cura a Carattere Scientifico G. Gaslini, Genova, Italy; 3 Dipartimento di Pediatria G. de Toni, Università degli Studi di Genova, Genova, Italy

## Abstract

**Objective:**

To investigate concordance between physicians and parents in rating the degree of functional ability of children with juvenile idiopathic arthritis (JIA).

**Methods:**

The attending physician and a parent were asked to rate independently the level of physical functioning of 155 patients with disease duration ≥ 5 years on a 6-point scale ranging from 1 = no disability (i.e. the child can do without difficulty all activities that children of his/her age can do) to 6 = severe disability (i.e. all activities are difficult for the child). At study visit, measures of JIA activity and damage were assessed. Agreement was evaluated with weighted kappa (<0.40 = poor agreement; 0.41–0.60 = moderate agreement; 0.61–0.80 = substantial agreement; >0.80 excellent agreement). Physician/parent evaluations were divided in 3 groups: 1) concordance; 2) parent over-rating = parent assessment over-rated relative to physician assessment; 3) physician over-rating = physician assessment over-rated relative to parent assessment. Factors affecting concordance/discordance were evaluated by means of Kruskal-Wallis or Chi-square/Fisher exact test.

**Results:**

Concordance, parent over-rating and physician over-rating were observed in 107 (69%), 29 (18.7%) and 19 (12.3%) evaluations, respectively. Kappa value was 0.69. Parent over-rating was associated with greater intensity of pain (p = 0.01) and higher Childhood Health Assessment Questionnaire (C-HAQ) score (p = 0.004), whereas physician over-rating was associated with more severe joint disease (p = 0.04 to <0.001), higher C-reactive protein (p = 0.03) higher frequency of Steinbrocker functional class = II (p < 0.001), and greater articular damage, as measured with the Juvenile Arthritis Damage Index (p < 0.001).

**Conclusion:**

Physicians and parents revealed fair concordance in rating functional ability of children with JIA. Parent over-rating was associated with greater child's pain and worse C-HAQ score, whereas physician over-rating was associated with greater severity of joint inflammation and damage.

## Introduction

The assessment of functional ability is of primary importance in the clinical evaluation of children with juvenile idiopathic arthritis (JIA). Physical disability is a central domain of disease outcome as prevention of loss of function is one of the main aims of JIA treatment. In the past, the level of physical functioning was usually determined through the use of physician-centered measures, such as the Steinbrocker functional classification [1]. Starting in the 90s, parent-centered measures, such as parent-proxy report of physical functioning with questionnaires [2-8], achieved increasing popularity. Most of the recent long-term outcome studies in JIA (reviewed in 9 and 10) have incorporated both physician-centered and parent-centered functional ability measures. However, little is known about the extent to which physicians' and parents' reports of physical functioning agree. In all chronic conditions, the parent's and patient's expectations and definition of disease severity do not often coincide with those of the professionals caring for their children. We previously found that a sizable proportion of parents either under or overestimate the degree of their children's functional ability, as measured with the C-HAQ, when compared with the objective physician's assessment [11].

The agreement on defining the level of functional ability of children with JIA is an important aspect of physician-parent interaction in clinical practice. Since the improvement in physical function is of foremost importance to parents and the patient, and the achievement of a normal functional ability status may have major prognostic implications, it is important to ascertain whether parents' and clinicians' opinions converge or diverge and to identify the factors that may explain the discordance. Substantial disagreement between parents and physicians over physical function can lead to difficulty in assessing the effectiveness of therapy or in evaluating the need for additional interventions. Furthermore, poor clinician/parent concordance may lead to parent dissatisfaction and decreased compliance.

In the present study, we have investigated the agreement between physicians and parents in the assessment of functional ability of children with JIA and attempted to identify factors affecting discordance.

## Methods

### Patient selection

All consecutive patients seen at the study unit between September, 2002 and June, 2004 who had JIA by the International League of Associations for Rheumatology (ILAR) criteria [12] and had at least 5 years of disease duration were included in the study. Due to their peculiar clinical features, particularly the presence of enthesitis and reduced spinal mobility, patients with enthesitis-related arthritis (ERA) or juvenile ankylosing spondylitis were excluded. A further reason for exclusion was the lack of instruments specifically validated for use in these conditions. The study protocol was approved by the Ethic Committee of the Istituto Gaslini of Genova, Italy

### Assessment of functional ability

The attending physician (AR or SV) and one parent (the mother, whenever available) were asked to rate independently the level of the child's functional ability on a 6-point categorical scale [13]. The question stem, "Considering the child's ability to do the activities of daily life, overall which describes he/she best?", prompted the respondent to choose from 6 response categories: 1 = no disability (i.e. the child can do all activities other children of his/her age can do with no problems); 2 = mild disability (i.e. the child can do almost all activities other children of his/her age can do with no problems); 3 = mild-to-moderate disability (the child can do most activities, but some activities are hard for him/her); 4 = moderate disability (the child can do most activities, but many activities are hard for him/her); 5 = moderate-to-severe disability (most activities are hard for the child); 6 = severe disability (all activities are hard for the child). The parents were instructed to consider the week before the study visit. The attending physicians were asked to base their judgment on their clinical impression at the time of the study visit. Beside the traditional physician-centered measures (see below), the physician assessed the ability of the child to perform some "core" functional activities (i.e. walking on flat ground and on tiptoes, squatting down, bending down, etc.) to get a general idea of the child's functional ability. Since all patients had long-standing disease, most of them were well known to the attending physician. Both attending physicians were pediatric rheumatologists with > 15 years' experience.

The same parent who provided the categorical rating of functional ability was also asked to complete the Italian version of the Childhood Health Assessment Questionnaire (C-HAQ) [14] (0 = best; 3 = worst). A second physician (EP or NS), who watched the clinical assessment of the attending physician, assigned independently the Steinbrocker functional classification [1]. Both these physicians were pediatric residents with > 2 years of training in pediatric rheumatology. Prior to the study initiation, they were instructed on the general clinical meaning and the scoring method of the Steinbrocker functional classification.

### Clinical assessment

Patient general information included onset age, sex, and ILAR category. The following clinical measures of JIA activity were recorded at the study visit: physician's global assessment of the overall disease activity measured on a 10-cm visual analogue scale (VAS) (0 = no activity; 10 = maximum activity); parent's global assessment of the child's well-being on a 10-cm VAS (0 = very good; 10 = very poor); parent's rating of the intensity of the child's pain (0 = no pain; 10 = maximum pain); count of joints with swelling, pain on motion/tenderness, restricted motion, and active arthritis [15]; duration of morning stiffness; erythrocyte sedimentation rate (ESR) (Westergren method); C-reactive protein (CRP) (nephelometry). The amount of articular and extra-articular damage was measured through the articular and extra-articular components of the Juvenile Arthritis Damage Index (JADI-A and JADI-E, respectively) [16]. Briefly, the JADI-A assessed 36 joints or joint groups for the presence of damage and the damage observed in each joint is scored on a 3-point scale (0 = no damage; 1 = partial damage; 2 = severe damage, ankylosis, or prosthesis). The maximum total score is 72. The JADI-E includes 13 items in 5 different organ/systems. Each item is scored as 0 or 1 if damage is absent or present, respectively, except for damage in each eye, which is scored on a 0–3 scale. The maximum total score is 17. The child's health-related quality of life (HRQL) was assessed by the same parent who provided the categorical rating of functional ability through the Italian version of the Child Health Questionnaire (CHQ) [14]. Briefly, the CHQ is a generic health instrument designed to capture the physical and psychosocial functioning of children 5 years of age and older. It includes 50 items/questions and yields two summary scores, the physical score (PhS) and psychosocial score (PsS). The mean ± SD norm based score from cross-cultural general populations for both PhS and PsS is 50 ± 10, with higher scores indicating better health.

### Radiographic assessment

Radiographic joint damage was scored according to the Poznanski method [17,18]. Briefly, this method is based on the measurement of the radiometacarpal length (RM) and of the length of the second metacarpal bone (M2). For each wrist, the number of standard deviations (SD) between the expected and the observed RM for the measured M2 was calculated according to the formulae reported by Poznanski et al [17]. The RM/M2 ratio, which constitutes the Poznanski score, reflects the amount of radiographic damage in the wrist. The more negative the Poznanski score is, the more severe the radiographic damage. In each patient, the Poznanski score was expressed as the mean of the 2 wrists.

### Statistics

Descriptive statistics were reported in terms of means, standard deviations, medians and upper and lower quartiles for continuous variables and in terms of absolute frequencies and percentages for categorical variables. Agreement between physicians and parents was evaluated with weighted kappa (<0.40 = poor agreement; 0.41–0.60 = moderate agreement; 0.61–0.80 = substantial agreement; >0.80 excellent agreement) [19]. Physician/parent evaluations were divided in 3 groups: 1) concordance = parent assessment equal to physician assessment; 2) parent over-rating = parent assessment over-rated relative to physician assessment; 3) physician over-rating = physician assessment over-rated relative to parent assessment. Comparison of quantitative variables among concordance/discordance groups was made by means of the non-parametric analysis of variance (Kruskal-Wallis test); the Dunn test was chosen as *a posteriori *test to assess the statistical significance of differences between pairs of patient groups. Comparison of qualitative data was performed by means of the χ^2 ^test, or the Fisher Exact test in case of expected frequencies less than 5. Bonferroni adjustment was applied as a correction for multiple comparisons to explore post-hoc differences between pairs of patients groups. All statistical tests were two sided; a P value of less than 0.05 was considered as statistically significant. The statistical package used was the "Statistica" (StatSoft Corp., Tulsa, OK).

## Results

A total of 155 patients, 35 males and 120 females, were included in the study. The ILAR category was systemic arthritis in 19 patients, rheumatoid factor (RF)-negative polyarthritis in 28 patients, RF-positive in 5 patients, persistent oligoarthritis in 42 patients, extended oligoarthritis in 41 patients, psoriatic arthritis in 5 patients, and undifferentiated arthritis in 15 patients. One-hundred-eighteen patients were antinuclear antibody-positive. All but 3 patients were of Italian ancestry. The much greater proportion of girls in our cohort is explained by the high prevalence of the ANA-positive subset of JIA in the Italian population. The main demographic and clinical features of the study patients are reported in Table 1. The values of the categorical disability scores are presented in table 2. None of the approached families declined to participate in the study. According to inclusion criteria, all patients had long-standing disease, with a minimum and median disease duration of 5 and 7.3 year, respectively. The median number of actively inflamed and functionally restricted joints (2 and 1, respectively) reflected moderate-to-low disease activity and severity. The median C-HAQ score of 0.3 meant that most children had mild disability. This is also reflected by the fact that only 3.9% of the patients were in Steinbrocker functional class III or IV and that 5% or less of the patients were in physician's or parent's categorical scale IV to VI.

**Table 1:** Demographic and clinical features of the 155 study patients

	N	Mean (SD)	Median	Lower Quartile	Upper Quartile
Age at disease onset, years	155	4.25 (3.2)	3.1	1.7	5.8
Age at study visit, years	155	12.5 (4.4)	11.8	9.2	14.9
Disease duration, years	155	8.5 (3.5)	7.3	5.8	10
Physician's global assessment^§^	153	3.6 (3.4)	2.5	0.5	6.3
Parent's global assessment^§^	148	1.79 (2.3)	0.65	0	3
Parent's pain assessment^§^	145	1.91 (2.4)	1	0	3
Swollen joint count	155	2.8 (5.1)	1	0	3
Tender joint count	155	3.1 (4.6)	1	0	4
Restricted joint count	155	4.9 (10.2)	1	0	5
Active joint count	155	3.9 (6.2)	2	1	5
C-HAQ score^$^	152	0.3 (0.5)	0.1	0	0.4
Morning stiffness, minutes	146	14.6 (35)	0	0	10
ESR, mm/hour^₤^	145	20.8 (17.1)	16	10	27
C-reactive protein, mg/dl^^^	146	0.7 (1.4)	0.1	0.1	0.8
JADI-Articular score°	155	2.3 (5.5)	0	0	2
JADI-Extra-articular score^+^	155	0.6 (1.2)	0	0	1
Poznanski score, units^&^	84	- 1.7 (2)	- 1.2	- 3.2	0.1
CHQ-Physical summary score^#^	119	49.7 (7.6)	52.6	47.2	55
CHQ-Psychosocial summary score^#^	119	48.9 (7.5)	49.1	43	54.3

C-HAQ: Childhood Health Assessment Questionnaire; ESR: erythrocyte sedimentation rate; JADI: Juvenile Arthritis Damage Index; CHQ: Child Health Questionnaire.

^§^On a 0–10 cm visual analogue scale (0 = best; 10 = worst);^$^Range of 0 (best) to 3 (worst);^₤^normal <15 mm/hour; ^normal <0.1 mg/dl;°range of 0 (best) to 72 (worst);^+^range of 0 (best) to 17 (worst);^&^abnormal < – 2 units;^#^mean ± SD norm based score for both physical and psychosocial scores: 50 ± 10.

**Table 2:** Results of categorical assessments of functional ability

		N (%)
Steinbrocker class, no. (%)	I	97 (62.6)
	II	52 (33.5)
	III	5 (3.3)
	IV	1(0.6)
Physician's categorical scale	I	95 (61.3)
	II	31 (20)
	III	22 (14.2)
	IV	2 (1.3)
	V	4 (2.6)
	VI	1 (0.6)
Parent's categorical scale	I	86 (55.5)
	II	34 (22)
	III	27 (17.4)
	IV	6 (3.9)
	V	1 (0.6)
	V I	1 (0.6)

Concordance, parent over-rating and physician over-rating were observed in 107 (69%), 29 (18.7%) and 19 (12.3%) evaluations, respectively. The weighted kappa value was 0.69, meaning substantial agreement. Table 3 shows the comparison of demographic and clinical variables and measures of disease severity in the 3 discordance groups. The parent over-rating group included relatively less females (p = 0.02). There was a relatively greater proportion of patients with persistent oligoarthritis in the concordance group and of patients with polyarthritis in the physician over-rating group (p = 0.005). Parent over-rating was associated with greater intensity of pain (p = 0.01) and higher CHAQ score (p = 0.004), whereas physician over-rating was associated with greater severity of joint disease, as reflected by greater frequency of polyarticular involvement (i.e. ≥ 5 affected joints) and worse joint counts (p = 0.04 to <0.001), higher CRP (p = 0.03), higher frequency of Steinbrocker functional class = II (p < 0.001), and greater articular damage, as measured with the JADI (p < 0.001).

**Table 3:** Factors affecting concordance between physicians' and parents' assessment of functional ability. Values are medians (interquartile range) unless otherwise indicated

	Concordance (N = 107)	Parent over-rating (N = 29)	Physician over-rating (N = 19)	P
No. (%) female	89 (83.2)	17 (58.6)	14 (73.7)	0.02^§^
ILAR category, no. (%)^$^				
Oligoarthritis persistent	41 (38.3)	6 (20.7)	4 (21)	0.005^§^
Oligoarthritis extended	35 (32.7)	9 (31)	3 (15.8)	
Polyarthritis	20 (18.7)	5 (17.2)	8 (42.1)	
Systemic arthritis	7 (6.5)	8 (27.6)	4 (21.1)	
Psoriatic arthritis	4 (3.8)	1 (3.5)	0 (0)	
ANA positive, no. (%)	85 (79.4)	19 (65.5)	14 (73.7)	0.25^§^
Patients with = 5 joints involved	31 (29.0)	13 (44.8)	15 (78.9)	<0.001^₤^
Age at onset, years	3 (1.7–5.5)	3.1 (1.66–6)	3.1 (1.68–6.58)	0.86
Age at visit, years	11.87 (9.7–14.5)	10.8 (8.9–14.6)	15 (9–19.6)	0.39
Disease duration, years	7.65 (5.63–10.1)	6.9 (5.19–8.8)	9.3 (6.2–13.4)	0.14
Physician's global assessment	2.4 (0–5.8)	2.5 (1.4–6.3)	5 (1–8.3)	0.23
Parent's global assessment	0.50 (0–3)	1.45 (0.45–3.55)	0.65 (0.45–3.7)	0.11
Parent's pain assessment	0.6 (0–2.5)	2 (0.55–5.2)	1.2 (0–3)	0.011
Swollen joint count	1 (0–3)	2 (1–3)	3 (0–11)	0.036
Tender joint count	1 (0–3)	2 (1–4)	4 (2–12)	<0.001
Restricted joint count	1 (0–3)	2 (0–5)	5 (2–18)	<0.001
Active joint count	1 (0–3)	2 (1–5)	6 (2–16)	<0.001
Morning stiffness, minutes	0 (0–5)	0 (0–10)	0 (0–10)	0.41
ESR, mm/hour	14 (10–27)	21 (10–35)	16 (10–21)	0.29
C-reactive protein, mg/dl	0.1 (0.1–0.58)	0.1 (0.1–1.44)	1.16 (0.1–1)	0.027
JADI-Articular score	0 (0–1)	1 (0–2)	3 (0–12)	<0.001
JADI-extraarticular score	0 (0–1)	0 (0–1)	0 (0–1.5)	0.29
Poznanski score, units (N = 84)	- 1.18 (- 2.8–0.095)	- 1.7(-3.1 – -0.05)	- 1.1 (-04 – -0.47)	0.69
CHQ-PhS	52.91 (47.26–55.28)	51.4 (44.4–52.9)	50.9 (42.4–53.9)	0.19
CHQ-PsS	49.95 (43.62–54.44)	47.6 (42.5–51.2)	50.3 (43–58.3)	0.23
C-HAQ score	0 (0–0.25)	0.3 (0–0.93)	0.1 (0–0.75)	0.004
Steinbrocker class, no (%)				
I	74 (69.2)	20 (69)	3 (15.8)	<0.001^₤^
II-IV	33 (30.8)	9 (31)	16 (84.2)	
Physician's categorical scale				
I	73 (68.2)	22 (75.9)	0 (0.0)	< 0.001^§^
II	17 (15.9)	4 (13.8)	10 (52.6)	
III-VI	17 (15.9)	3 (10.3)	9 (47.4)	
Parent's categorical scale				
I	73 (68.2)	0 (0.0)	13 (68.4)	< 0.001^§^
II	17 (15.9)	15 (51.7)	2 (10.5)	
III-VI	17 (15.9)	14 (48.3)	4 (21.1)	

See Table 1 for abbreviations and score ranges. ^$^For purposes of the analysis, patients with the ILAR category of undifferentiated arthritis (n = 15) were classified within polyarthritis and extended oligoarthritis categories based on the number of joints affected in first 6 months of disease.

Comparisons for all quantitative variables were made with the Kruskall-Wallis test. ^₤^Chi-square test; ^§^Fisher Exact test.

## Discussion

In the present study, we found an overall fair agreement between the physicians and the parents in rating the level of functional ability of children with JIA, with more than two third of the evaluations being concordant. The weighted kappa value fell in the "substantial agreement" range. These findings are surprising because parents and physicians were measuring something different: the parent's rating was supposed to reflect the child's average performance over the preceding week, whereas the physicians were assessing the child at one point in time only. Furthermore, the parents were likely to base their judgment on the direct observation of the child's ability to perform activities in daily life, whereas the physicians had to rely on the findings of clinical assessment. The concordance seen in this study compares favorably with the level of agreement previously observed for other health assessments, such as C-HAQ disability, pain, disease activity, and disease remission [11,20-22]. In a past study that compared parent-proxy reported to physician-observed assessment of functional ability, as measured with the C-HAQ, we found that for only 43% of patients there was concordance [11]. In another analysis, we observed only a moderate agreement between parents and physicians in rating the intensity of children's pain [20]. When we investigated the discrepancy between physician's and parent's global assessments of disease status, we found that physicians and parents may perceive the health status of children with JIA differently, with parents providing more frequently lower rating [21]. We recently observed a frequent discordance between physicians and parents in rating the disease as inactive disease in children with JIA [22]. This indicated that a number of instances of physician-defined inactive disease may not be agreed upon by the parents. The greater agreement obtained with the administration of a 6-point categorical scale suggests that the use of simpler instruments may facilitate physician-parent concordance. However, the scale used in the study is not intended as a substitute for the more detailed and comprehensive instruments, such as the C-HAQ, which give the best information regarding children's functional ability. Furthermore, it should be acknowledged that the validity and reliability of the instrument used in the study were not formally assessed.

Discordance between the physicians and the parents was observed for 31% of patients. Among discordant evaluations, parent's over-rating relative to physician's assessment (i.e. the parent judged the level of child's physical function as worse than the physician) was seen more frequently than physician's over-rating relative to parent's assessment (i.e. the physician judged the level of child's physical function as worse than the parent). Parents judged child's functional ability as worse than physicians with greater intensity of the child' pain and with higher CHAQ score. Physicians judged child's functional ability as worse than the parents with greater severity of joint disease, higher CRP, higher frequency of Steinbrocker functional class ≥ II, and greater articular damage, as measured with the JADI. These findings suggest that the presence of pain may have a relevant influence on proxy-reported functional ability, leading the parents to potentially emphasize their child's disability. The relationship between the parents' categorical assessment and the C-HAQ score was expected because this questionnaire is a proxy-reported measure of the child's functional ability. That physicians' overestimation was associated with the physician-centered measures of joint inflammation or damage suggests that physicians tends to intuitively adjust the amount of impairment that they see to the objective signs of disease activity and joint impairment presented by the patient.

We must acknowledge that by describing the parent-physician discordance in rating the level of functional ability of children with JIA, we cannot imply that the physician's assessment is the right one. It is well known that parents and doctors may have widely different perspectives relating to their beliefs about health and illness, their expectations of medical care, their priorities for treatment and the ways in which they interpret information about child's disease [11]. It is likely that many other factors not primarily related to disease activity, such as psychosocial issues, may have a major influence on the parent's perception of the child's well-being [23,24]. We asked the mothers to rate the health status of their children, but did not obtain information on children's self reporting. However, using only parent's proxy reports instead of both parent's and patient's self reports would fail to capture that parents and children may differ in their perception of health [20,25]. A wide variation in agreement between adolescents with JIA and their parents about physical health, functional ability, and HRQL was recently reported [26]. Notably, the accuracy of the parents in reporting their children's functional ability lessens as children become adolescents and the parents no longer observe some activities. The low level of disease activity and disability in most of our patients may have limited the generalizability of our study. In addition, it might have facilitated the achievement of a concordance between raters. However, our patients represent a consecutive sampling of our clinic population and are likely representative of the patients seen in most tertiary pediatric rheumatology centers. It should be, recognized, however, that the exclusion of patients with ERA, which represent around 6% of our JIA population, may limit the generalizability of our findings. The much greater prevalence of females in our cohort might also affect the generalizability of our results.

In conclusion, we found that physicians and parents revealed an overall fair concordance in rating functional ability of children with JIA. Parent over-rating was associated with greater intensity of child's pain and worse C-HAQ disability, whereas physician-over rating was associated with greater severity of joint inflammation and damage.

## Authors' contributions

AR conceived the study, and participated in its design and coordination, and in manuscript preparation. NR, SV and AM participated in the design of the study, and in the analysis and interpretation of data. AM participated in manuscript preparation. EP, NS, CM, SV, AB and AL participated in the acquisition of data. AP performed the statistical analysis. All authors have read and approved the final manuscript.
